# Resting heart rate, physiological stress and disadvantage in Aboriginal and Torres Strait Islander Australians: analysis from a cross-sectional study

**DOI:** 10.1186/s12872-016-0211-9

**Published:** 2016-02-11

**Authors:** Alice Zhang, Jaquelyne T. Hughes, Alex Brown, Paul D. Lawton, Alan Cass, Wendy Hoy, Kerin O’Dea, Louise J. Maple-Brown

**Affiliations:** Menzies School of Health Research, Charles Darwin University, Darwin, Australia; School of Medicine, University of Maryland, Baltimore, USA; Division of Medicine, Royal Darwin Hospital, Darwin, Australia; South Australian Health and Medical Research Institute, Adelaide, Australia; School of Population Health, University of South Australia, Adelaide, Australia; Centre for Chronic Disease, The University of Queensland, Brisbane, Australia

**Keywords:** Heart rate, Socioeconomic status, Stress, Indigenous, Australia

## Abstract

**Background:**

Lower socioeconomic status has been linked to long-term stress, which can manifest in individuals as physiological stress. The aim was to explore the relationship between low socioeconomic status and physiological stress in Aboriginal and Torres Strait Islander Australians.

**Methods:**

Using data from the eGFR Study (a cross-sectional study of 634 Indigenous Australians in urban and remote areas of northern and central Australia), we examined associations between resting heart rate and demographic, socioeconomic, and biomedical factors. An elevated resting heart rate has been proposed as a measure of sustained stress activation and was used as a marker of physiological stress. Relationships were assessed between heart rate and the above variables using univariate and multiple regression analyses.

**Results:**

We reported a mean resting heart rate of 74 beats/min in the cohort (mean age 45 years). On multiple regression analysis, higher heart rate was found to be independently associated with Aboriginal ethnicity, being a current smoker, having only primary level schooling, higher HbA1c and higher diastolic blood pressure (model R^2^ 0.25).

**Conclusions:**

Elevated resting heart rate was associated with lower socioeconomic status and poorer health profile in Aboriginal and Torres Strait Islander Australians. Higher resting heart rate may be an indicator of stress and disadvantage in this population at high risk of chronic diseases.

**Electronic supplementary material:**

The online version of this article (doi:10.1186/s12872-016-0211-9) contains supplementary material, which is available to authorized users.

## Background

It is well established that Indigenous Australians are one of the most disadvantaged groups in Australia [1, 2]. The most recent estimates of the gap in life expectancy between Indigenous and non-Indigenous Australians are twelve and ten years for men and women respectively [1]. Disadvantage is evident across the full range of socioeconomic indicators including educational attainment, employment, income and house crowding [1]. In addition, almost one half of Indigenous Australians have a disability or long-term health condition that affects their health and general well-being [1].

Poor health is strongly associated with low socioeconomic status (SES). Individuals of low socioeconomic status experience poorer living conditions and reduced access to resources and services [3]. Disadvantage can cause long-term or chronic stress [4]. Continuing anxiety relating to low income, insecurity of employment or housing tenure, social isolation and lack of control over one’s working environment and home life, cause chronic stress and powerfully affect health [5]. It has been suggested that a greater number of stressors leads to the expectation of negative outcomes, which may contribute to hopelessness, an inability to cope, and increased vulnerability to future stressors [3, 6]. As a result, low SES can act as and be considered a composite marker of chronic psychological stress [7].

The body reacts to stressful stimuli by activating its physiological systems that serve to adapt to imminent challenges and maintain homeostasis. However, sustained or repeated exposure to stress, recurrent physiological and dysregulated adaptive responses can be harmful, resulting in a biological “wear and tear,” which has been referred to as allostatic load [8]. While multiple systems are affected, the cardiovascular system is one of the most susceptible [9, 10]. The sympathetic nervous system (SNS), hypothalamic pituitary adrenal (HPA) axis, and renin-angiotensin system release stress hormones in response to stressful stimuli, many of which can induce heightened cardiovascular reactivity. An elevated resting heart rate has been proposed as one indicator of sustained stress activation [11, 12]. Resting heart rate has been shown to be correlated with heart rate variability, another marker for sympathetic innervation that has been linked with chronic stressors [13]. Increased levels of worry, which has been used in previous studies to represent continuous stress, have been associated with an elevated resting heart rate [14]. In addition, studies measuring change in allostatic load over time have included a high resting heart rate as a component [15, 16].

In the context of the relationship between physiological stress, socioeconomic status, and health, our aim was to explore associations between resting heart rate (a parameter that is routinely measured in clinical practice) and demographic, biomedical and socioeconomic factors. We examined these associations in a cross-sectional cohort of 634 Indigenous Australians from urban and remote regions of northern and central Australia. Our hypothesis was that Indigenous Australians would demonstrate associations between markers of disadvantage and physiological markers of stress, as measured by resting heart rate, independent of biological markers of cardiovascular risk.

## Methods

Data was collected as part of the baseline phase of the eGFR Study: Accurate Assessment of Kidney Function in Indigenous Australians, which was conducted from 2007 to 2011 [17].

### Participant characteristics and recruitment

655 Indigenous participants were recruited from 4 geographical regions in Australia: Top End, Central Australia, Western Australia (Gold Fields and Kimberley regions), and Far North Queensland (Cairns and Torres Strait). All participants were aged 16 years and above. As per the definition used by the Australian National Census, participants were: (i) of Aboriginal and/or Torres Strait Islander descent, (ii) self-identified as Aboriginal and/or Torres Strait Islander, and (iii) accepted as such by the community in which they lived. Participants were recruited across five strata of health, based on eGFR and co-existing health conditions (diabetes, hypertension, chronic kidney disease, albuminuria). Individuals who were on dialysis, with rapidly changing kidney function, pregnant or breastfeeding, or with an allergy to iodine-based contrast media were excluded from the study. Informed consent was provided via a face-to-face discussion, with an interpreter available if required, and obtained using the NHMRC Guidelines for Ethical Conduct in Aboriginal and Torres Strait Islander Health Research [17]. Participants were recruited through participating primary health care services or hospital outpatient/outreach clinics, as well as through community networks, word-of-mouth and self-referral.

Of the 655 participants recruited to the study, 634 participants were included in the analysis as they had data for at least two heart rate measurements.

### Assessment of reference GFR and other clinical measures

Methods have been previously described in detail [17]. In brief, blood and urine samples were sent to local pathology laboratories to determine urine albumin-creatinine ratio (ACR) and HbA1c. Reference glomerular filtration rate (mGFR) was determined by injecting non-isotopic iohexol into an antecubital vein and measuring the renal clearance of iohexol over four hours [18]. Microalbuminuria was defined as urine ACR ≥2.5 and ≤ 25 mg/mmol in men and ≥3.5 and ≤ 25 mg/mmol in women. Macroalbuminuria was defined as ACR > 25 mg/mmol [19].

Blood pressure and resting heart rate were obtained after the participant had been resting for at least 5 min in a clinic setting. Resting heart rate is sensitive to measurement conditions [20]. As such, we obtained three resting measurements spaced 2 min apart in the same environment. These measurements were taken using a Welch Allyn blood pressure machine. Measurements taken by automated blood pressure machines have been found to correlate with manual readings [21].

The social history questionnaire included information on:

1. Demographic variables: age, gender, ethnicity, ethnic admixture, region participant identifies as coming from, primary language spoken at home, time in current location, number of times moved in the last 5 years, number of bedrooms in the house, total number of occupants in the house usually, and last night, and number of those aged ≤ 15 years;
2. socioeconomic characteristics: housing tenure, landlord type if renting, highest level of school completed, highest qualification, employment status, ever worked, main income source, gross household income;

Ethnic admixture was defined as the number of Indigenous grandparents the participant reported having. Equivalised income, which accounts for economies of scale in a household, was calculated by dividing the midpoint of each income category by the weighted sum of household members. The weighted sum was calculated as per a modified Organisation for Economic Co-operation and Development (OECD) scale, in which the first adult aged 15 years and above is weighted as 1, every other adult as 0.5, and every child as 0.3 [22]. The number of occupants per bedroom was calculated by dividing the number of occupants usually in the household by number of bedrooms in the house.

### Statistical analysis

Stata version 12 (Stata Corporation, College Station, TX) was used for all analysis. Data are presented as mean (standard deviation), number (frequency) and median (range). Variables were assessed for normal distribution and log transformed when required; only urine ACR was log transformed. The biomedical, social and socioeconomic variables noted above were compared with the outcome variable (mean resting heart rate) in simple and multiple regression models. The outcome variable was represented as a continuous variable. For predictor variables with more than two categories, the reference group was set to the largest participant number for the social variables and the highest SES for the socioeconomic variables.

Multiple linear regression analysis was performed to determine independent relationships between biomedical, social and socioeconomic variables and the dependent variable resting heart rate. Covariates included in this analysis consisted of all variables with a significant bivariate correlation with heart rate (*p* ≤ 0.05). The following variables were excluded from the final model due to correlations with other independent variables: number of bedrooms, landlord type and time in current location. “Not reported” categories were included for highest qualification, employment and equivalised income variables due to the high numbers of participants for whom data was not reported. There were no significant interactions between age or gender and variables in the multiple regression analysis.

### Ethics approval

The eGFR study received approval from the joint Human Research Ethics Committee of Menzies School of Health Research and the Northern Territory Department of Health and Community Services. The Aboriginal sub-committee, which has absolute right of veto, and the main committee considered and approved the study. The study was also approved by: Central Australian Human Research Ethics Committee, Western Australia Aboriginal Health Information and Ethics Committee, Royal Perth Hospital Ethics Committee and Cairns and Hinterland Health Services District Human Research Ethics Committee.

## Results

Participant characteristics (*n* = 634) are presented in Table 1. Torres Strait Islander participants represented a substantial proportion (19.9 %) of the participants compared to the nationwide Torres Strait Islander proportion (6 %) [1]. Almost half of the participants (48.3 %) came from the Northern Territory (NT), with roughly equal representation from the Top End (25.3 %) and Central Australian (23 %) regions of the NT. The majority of participants reported four Indigenous grandparents. Most participants had completed Year 10 or higher in school, rented their home, hadn’t moved in the last five years, and received their income as salary or wages. The mean resting heart rate was 74 beats per minute (bpm).

**Table 1:** Participant Characteristics (*n* = 634)

Demographic, biomedical characteristics		
Data presented as mean ± SD		
Age (yr)		45 ± 15
Gender	Female	397
Ethnicity	Aboriginal	454
	Torres Strait Islander	125 (19.7)
	Both Aboriginal and Torres Strait Islander	55 (8.7)
Region From	Top End – NT	147 (25)
	Central Australia – NT	137 (23.3)
	Queensland (Northern and Torres Strait)	174 (29.6)
	Western Australia (Kimberley and Gold Fields)	130 (22.1)
Number of Indigenous Grandparents	1	32 (5.4)
	2	103 (17.4)
	3	73 (12.3)
	4	385 (64.9)
Current Smoker		262 (42)
Currently Treated with Beta Blockers		36 (5.5)
Heart Rate (beats per min)		74 ± 13
HbA1c (%)		6.7 ± 2
BMI (kg/m^2^)		29.9 ± 7.2
Waist (cm)		100 ± 16
Systolic BP (mmHg)		118 ± 18
Diastolic BP (mmHg)		74 ± 10
mGFR^a^ (mls/min/1.73 m^2^)		103 (82, 121)
mGFR stratum (mls/min/1.73 m^2^)	>90	396 (67.2)
	60–89	115 (19.5)
	30–59	52 (8.8)
	<30	26 (4.4)
Albuminuria	Normoalbuminuria	353 (58.9)
	Microalbuminuria	111 (18.5)
	Macroalbuminuria	135 (22.5)
Socioeconomic characteristics		
Data presented as n (%) or median (range)		
Times Moved in Last Five Years	0	311 (51.7)
	1–5	263 (43.8)
	6+	27 (4.5)
Time in Current Location (years)		5 (0–50)
Usual Number of Occupants		4 (0–33)
Number of Occupants Aged Under 15 Years		1 (0–18)
Number of Occupants per Bedroom		1.4 (0–16)
Number of Bedrooms in House	1	51 (8.2)
	2	98 (15.8)
	3	299 (48)
	4	130 (20.9)
	5	44 (7.1)
Housing Tenure	Own/Being Purchased	90 (14.4)
	Rent or Other Tenure	537 (85.6)
Landlord Type	Government/state/territory housing authority	207 (33.5)
	Employer	16 (2.6)
	Indigenous housing organization	186 (30.2)
	Private	13 (2.1)
	Other	71 (11.5)
	Not applicable	124 (20.1)
Highest Level of School Completed	Primary school or never went	83 (13.4)
	Year 7–9	142 (22.9)
	Year 10 or equivalent	249 (40.2)
	Year 12 or equivalent	146 (23.5)
Highest Qualification	No post-school qualification	289 (45.6)
	Trade/certificate/apprentice	293 (46.2)
	University degree or higher	36 (5.7)
	Not Reported	16 (2.5)
Employment	Fully employed	221 (34.9)
	Not fully employed and has worked	331 (52.2)
	Not fully employed and has never worked	30 (4.7)
	Not Reported	52 (8.2)
Main Income Source	Salary/wages	334 (57.4)
	Other	248 (42.6)
Equivalised income (per week)	$1–199	66 (10.4)
	$200–499	182 (28.7)
	$500+	122 (19.2)
	Not Reported	264 (41.6)

^a^Data presented as median (IQR – interquartile range)

Due to missing data, n varied for the following: *n* = 625, smoker; *n* = 610, HbA1c, waist; *n* = 633, BMI; *n* = 589, mGFR; *n* = 599, albuminuria

Microalbuminuria was defined as urine ACR ≥2.5 and ≤ 25 mg/mmol in men and ≥3.5 and ≤ 25 mg/mmol in women; macroalbuminuria defined as ACR > 25 mg/mmol

Results of bivariate correlations with heart rate are presented in the Additional file 1: Table S1. Aboriginal ethnicity, current smoking and housing tenure of renting were all associated with a higher heart rate. In addition, moving more than six times in the last five years and having no post-school qualification and an equivalised income of $200 to 499 each week were all significantly associated with a higher resting heart rate. A gradient of resting heart rate was observed for level of schooling completed, and employment status. Among the biomedical variables, HbA1c, urine ACR, mGFR and diastolic BP were significantly correlated with heart rate on bivariate analysis. There were no significant bivariate associations for the following variables with heart rate: usual number of household occupants, number of household occupants aged under 15 years, number of occupants per bedroom.

On multiple regression analysis (Table 2), the following variables explained approximately one quarter of the variance (R^2^ = 0.252) in heart rate: female gender; current smoker; having a schooling level below Year 10; HbA1c, diastolic BP, mGFR (mGFR 60-90mls/min/1.73 m^2^); and Torres Strait Islander participant (lower heart rate than Aboriginal participants). Diastolic BP was included, as opposed to systolic BP, because diastolic BP demonstrated a stronger association on univariate analysis. Results were unchanged after the exclusion of 36 participants currently treated with beta blockers.

**Table 2:** Multivariate regression model: associations between resting heart rate and demographic, socioeconomic, and biomedical variables (*n* = 516, R^2^ = 0.252)

		β Coefficient [95 % CI]	*P* value
Gender	Female	2.43 [0.37, 4.48]	**0.021**
Age		−0.14 [−0.24, −0.05]	**0.004**
Ethnicity	Aboriginal	--	--
	Torres Strait Islander	−6.4 [−9.17, −3.62]	**<0.001**
	Both Aboriginal and Torres Strait Islander	−2.43 [−5.93, 1.07]	0.173
Smoking Status	Current Smoker	3.48 [1.29, 5.67]	**0.002**
Times Moved in Last Five Years	0	--	--
	1–5	−1.09 [−3.23, 1.04]	0.314
	6+	2.11 [−3.04, 7.26]	0.421
Housing Tenure	Own or being purchased	--	--
	Rent or other tenure	1.22 [−1.90, 4.34]	0.443
Highest Year of School Completed	Primary school or never went	7.57 [3.31, 11.84]	**0.001**
	Year 7–9	4.01 [0.66, 7.36]	**0.019**
	Year 10 or equivalent	2.23 [−0.52, 4.98]	0.111
	Year 12 or equivalent	--	--
Highest Qualification	No post-school qualification	1.35 [−3.00, 5.71]	0.542
	Trade/certificate/apprentice	1.30 [−3.22, 5.83]	0.572
	University degree or higher	--	--
	Not Reported	−3.50 [−15.68, 8.68]	0.572
Equivalised Income	$1–199	−0.88 [−5.04, 3.28]	0.678
	$200–499	−0.23 [−3.42, 2.95]	0.886
	$500+	--	--
	Not Reported	−0.57 [−3.68, 2.55]	0.721
Employment	Fully Employed	--	--
	Not fully employed and has worked	−0.34 [−2.82, 2.15]	0.790
	Not fully employed and has never worked	1.21 [−4.10, 6.52]	0.654
	Not Reported	−3.49 [−7.97, 1.00]	0.127
HbA1c (%)		1.23 [0.60, 1.86]	**<0.001**
Diastolic BP (mmHg)		0.27 [0.17, 0.37]	**<0.001**
Urine ACR (mg/mmol)		0.20 [−0.40, 0.80]	0.515
mGFR (mls/min/1.73 m^2^)	>90	--	--
	60–89	−3.12 [−5.95, −0.29]	**0.031**
	30–59	0.46 [−3.85, 4.77]	0.834
	<30	−2.39 [−8.28, 3.50]	0.426

RMSE = 11.282, Prob > F = 0. P values were bolded if they were statistically significant (p < 0.05)

## Discussion

We examined associations between heart rate and demographic, socioeconomic and biomedical factors in a cross-sectional cohort of Indigenous Australians, a population with lower SES than the general Australian population. The following were independently associated with heart rate: HbA1c, diastolic BP, Aboriginal (rather than Torres Strait Islander) ethnicity, being a current smoker, and having only primary level schooling.

The reported mean heart rate of 74 bpm is consistent with previously reported heart rates for Indigenous Australians, which range from 72 to 77 bpm [23, 24]. However, the mean heart rate in the current study is higher compared to the reported heart rates of non-Indigenous Australians and Caucasians. Heart rates range from 60 to 65 bpm for healthy patients [25, 26] and 66 to 74 bpm for those with a variety of health conditions, including end-stage renal disease and coronary artery disease [24, 27]. There is increasing evidence that cardiovascular risk increases with resting heart rates greater than 60 bpm, after controlling for beta blockers and medication use [28, 29].

The observed associations between resting heart rate and several demographic, socioeconomic, and biomedical factors explained 25 % of the variance in heart rate on multiple regression analysis. Out of the biomedical factors noted to be independently associated with heart rate (HbA1c, diastolic BP, mGFR), higher blood pressure has consistently been reported to occur with an increased heart rate. These findings occurred in laboratory settings with simulated social stressors or were correlated with stressful work conditions [30–32]. However, there have been inconsistencies in the literature as to whether both or only one of systolic and diastolic blood pressures are associated. We observed an association between diastolic blood pressure and heart rate, which is consistent with previous study findings linking cardiovascular reactivity to social stressors [10, 11]. Our findings indicate an association between increased heart rate and being a current smoker, consistent with a previous report that linked heart rate variability with smoking status [33].

There are limited available data on the relationship between high resting heart rate with HbA1c and GFR. In particular, the observed relationship between heart rate and a GFR between 60 and 90 mls/min/1.73 m^2^ could not be explained adequately. The relationship is unlikely to have resulted from beta blocker medication, as sensitivity analysis performed excluding participants taking beta blockers did not alter the results. It is possible that those with a GFR between 60 and 90 mls/min/1.73 m^2^ have fewer cardiometabolic risks, while those with higher GFR values may be obese and exhibit renal hyperfiltration. However, it has been noted that a unified physiological response occurs among cardiovascular, immune and endocrine systems in response to stressors [32] and heart rate recovery has been associated with metabolic factors [34, 35].

In addition to the biomedical factors, our findings indicated that Aboriginal ethnicity was associated with an increased heart rate compared to Torres Strait Islander participants. However, it must be stressed that socioeconomic factors could account for the difference. Because Torres Strait Islander participants were primarily screened at the main economic centre in the Torres Strait, the cohort contained Torres Strait Islander participants with higher socioeconomic status. On the other hand, Aboriginal participants were from diverse remote and urban areas. In the entire cohort, we observed that heart rate was associated on bivariate analysis with housing tenure, schooling and qualifications, employment status and equivalised income; however, only schooling remained independently associated with heart rate on multivariate analysis. Individual income and education have been previously identified as one predictor of heart rate in adolescents in the United States [36]. An elevated resting heart rate has been observed with greater disadvantage, as measured by education level, employment, home ownership, and the ability to take holidays [37, 38].

It is possible that associations between lower socioeconomic status and heart rate result from higher level of stress. Low income and education levels can cause disadvantaged individuals to live in chronically stressful environments, with financial insecurity, negative life events, and emotional and cognitive factors as contributing factors [3, 4]. Previous hypotheses have stated that exposure to stress from low SES results in altered physiological responses, especially with regard to immune, neuroendocrine and cardiovascular functioning [39–41]. Not all measures of altered physiological responses were measured in the current study.

Considering the potential relationship between low SES and stress, the current study findings indicate that an elevated resting heart rate in Indigenous Australians could be related to stress resulting from low socioeconomic status. The clinical impact of these findings is that clinicians could view resting heart rate as not only a cardiovascular risk factor but also as a possible indicator of stress and disadvantage. Social disadvantage has been identified as one chronic stressor in Aboriginal communities, and their marginalization has been linked with ill health [42, 43]. The cumulative stress burden, in addition to low socioeconomic status, has also been reported to be associated with depression in Central Australian men [44]. The current study accounted for some of the components contributing to social disadvantage in Indigenous communities, such as housing tenure, education level, employment status, and income. Overcrowded, unstable and poor housing conditions not only impact the mental and physical health of its occupants but also create an unfavourable environment for children to grow up in. Children’s school performance can be negatively affected, which in turn influences their ability to obtain secure and skilled employment in the future. However, the factors affecting socioeconomic status are complex and interconnected. Reduced life expectancy, health outcomes, educational attainment, and employment opportunities can be affected by other circumstances, such as alcohol abuse, violence, or lack of adequate infrastructure within the community [45].

The current study includes the following limitations. Causality between stress and the previously discussed demographic, socioeconomic, and biomedical factors cannot be inferred because of the cross-sectional nature of the current study. In addition, while having a comparison group would have improved the interpretation of these findings, it was not practical in the current study. Furthermore, stress was not directly measured, as stress is a complex variable influenced by multiple interacting factors such as depression, anxiety, personality characteristics, and social support. It has been suggested that social participation and autonomy be used to measure stress [5]. However, because people interpret and react to stressful events differently, it can be advantageous to use physiological stress measurements. The analysis featured resting heart rate, which acts as a possible marker of an activated hypothalamic pituitary adrenal axis and an increased sympathetic nervous system. But resting heart rate is only one measurement of physiological stress and is commonly associated with experimental stress and psychological trauma [46]. In addition, the standard method for heart rate measurement is by electrocardiogram (ECG). It was not feasible to assess both heart rate variability and resting heart rate in the current study and we chose to assess resting heart rate as it is a routine clinical measurement. Finally, participants of the current study were volunteers from urban and remote areas in Northern Territory, Western Australia and Queensland, Australia. Participants are not representative of the source population or Indigenous Australians generally as participants were recruited based on specific strata related to kidney health. Nonetheless, our study included a vast geographical area in Australia and featured a large number of participants (*n* = 634) with a range of health and chronic diseases status.

## Conclusions

In conclusion, we report that an elevated resting heart rate was associated with demographic, socioeconomic and biomedical characteristics in Indigenous Australians. We postulate that stress arising from lower socioeconomic status could be a key contributor to the elevated heart rate. However, because our cohort was preselected based on renal function, our findings may not be applicable to the broader population. To further examine the relationship between stress and disadvantage in Indigenous Australians and the impact of chronic stress on health outcomes, we suggest a longitudinal study with detailed measurement of stress at baseline. Since stress is a complex variable that can be difficult to define and measure, it may be useful to include both qualitative and quantitative measurements of stress.

## Additional file

Additional file 1: Table S1. Univariate regression analysis of resting heart rate. Data are beta coefficient and 95 % confidence intervals. (DOCX 22 kb)
