# Supplementary material for: Resting heart rate, physiological stress and disadvantage in Aboriginal and Torres Strait Islander Australians: analysis from a cross-sectional study
Source: BMC Cardiovasc Disord. 2016 Feb 11;16:36. doi: 10.1186/s12872-016-0211-9 (PMC4751751; doi:10.1186/s12872-016-0211-9)
Supplement: Additional file 1: Table S1. — Univariate regression analysis of resting heart rate. Data are beta coefficient and 95 % confidence intervals. (DOCX 22 kb) [file 12872_2016_211_MOESM1_ESM.docx]

**Additional file 1:** **Table S1: Univariate regression analysis of resting heart rate.**

Data are beta coefficient and 95% confidence intervals.

|  | | | | **Mean Heart Rate** | **Coefficient**  **[95% CI]** | **P value** |
| --- | --- | --- | --- | --- | --- | --- |
| Age |  | | | | -0.054  [-0.12, 0.013] | 0.11 |
| Gender | Male | | | 73 | -- | Ref |
|  | Female | | | +1 | 1.33  [-0.72, 3.38] | 0.202 |
| Ethnicity | Aboriginal | | | 76 | -- | Ref |
|  | Torres Strait Islander | | | -9 | -9.03  [-11.45, -6.61] | **< 0.001** |
|  | Both Aboriginal and Torres Strait  Islander | | | -5 | -4.82  [-8.24, -1.40] | **0.006** |
| Smoking Status | Ex smoker or never smoked | | | 72 | -- | Ref |
|  | Current smoker | | | +4 | 4.18  [2.18, 6.17] | **< 0.001** |
| Number of Bedrooms in House | 1 | | | -2 | -2.17  [-5.87, 1.53] | 0.251 |
|  | 2 | | | -3 | -2.88  [-5.72, -0.04] | **0.047** |
|  | 3 | | | 75 | --- | Ref |
|  | 4 | | | -3 | -2.56  [-5.12, 0.01] | 0.051 |
|  | 5 | | | -5 | -5.24  [-9.19, -1.30] | **0.009** |
| Housing Tenure | Rent or other tenure | | | 74 | -- | Ref |
|  | Own or being purchased | | | -3 | 2.85  [0.03, 5.67] | **0.048** |
| Landlord Type | Government/state/territory housing | | | 75 | -- | Ref |
|  | Employer | | | -5 | -4.64  [-11.02, 1.75] | 0.154 |
|  | Indigenous housing organisation | | | 0 | 0.72  [-1.76, 3.21] | 0.568 |
|  | Private | | | 0 | 0.31  [-6.73, 7.34] | 0.931 |
|  | Other | | | -6 | -5.50  [-8.88, -2.11] | **0.001** |
|  | Not applicable | | | -2 | -1.89  [-4.68, 0.91] | 0.185 |
| Highest Year of School Completed | Primary school or never went | | | +6 | 5.62  [2.24, 9.01] | **0.001** |
|  | Year 7-9 | | | +6 | 6.04  [3.14, 8.95] | **< 0.001** |
|  | Year 10 or equivalent | | | +3 | 3.46  [0.89, 6.03] | **0.008** |
|  | Year 12 or equivalent | | | 70 | -- | Ref |
| Highest Qualification | No post-school qualification | | | +6 | 5.48  [1.09, 9.88] | **0.015** |
|  | Trade/certificate/apprentice | | | +4 | 3.84  [-0.56, 8.23] | 0.087 |
|  | University degree or higher | | | 69 | -- | Ref |
|  | Not Reported | | | +3 | 3.11  [-4.36, 10.59] | 0.414 |
| Equivalised Income | $1-199 | | | +3 | 2.27  [-1.54, 6.08] | 0.243 |
|  | $200-499 | | | +4 | 3.13  [0.21, 6.04] | **0.036** |
|  | $500+ | | | 71 | -- | Ref |
|  | Not Reported | | | +3 | 2.37  [-0.36, 5.09] | 0.089 |
| Employment | Fully Employed | | | 72 | -- | Ref |
|  | Not fully employed and has  worked before | | | +3 | 2.86  [0.71, 5.01] | **0.009** |
|  | Not fully employed and has never  worked before | | | +6 | 6.01  [1.20, 10.82] | **0.014** |
|  | Not Reported | | | -2 | -1.71  [-5.53, 2.10] | 0.377 |
| Times Moved in Last Five Years | 0 | | | 74 | -- | Ref |
|  | 1-5 | | | -1 | -0.89  [-2.96, 1.18] | 0.398 |
|  | 6+ | | | +6 | 6.92  [1.96, 11.88] | **0.006** |
| Time in Current Location | | |  | | -0.18  [-0.31, -0.05] | **0.008** |
| HbA1c (%) | | |  | | 0.99  [0.43, 1.55] | **0.001** |
| Urine ACR (mg/mmol) | | |  | | 0.75  [0.29, 1.22] | **0.002** |
| BMI (kg/ m^2^) | | |  | | 0.04  [-0.09, 0.18] | 0.525 |
| Waist (cm) | | |  | | 0.04  [-0.02, 0.10] | 0.198 |
| Systolic BP (mmHg) | | |  | | 0.05  [-0.01, 0.10] | 0.083 |
| Diastolic BP (mmHg) | | |  | | 0.31  [0.22, 0.41] | **< 0.001** |
| mGFR (mls/min/1.73m^2^) | | > 90 | | 74 | -- | Ref |
|  |  | 60-89 | | -3 | -3.20  [-5.86, -0.54] | **0.019** |
|  |  | 30-59 | | +1 | 0.62  [-3.09, 4.32] | 0.744 |
|  |  | < 30 | | -1 | -0.74  [-5.82, 4.35] | 0.776 |
